# Age Adaption and Psychometric Properties of the Swedish Version of the Four-Components and Stimulus Module from the Index of Dental Anxiety and Fear (IDAF) for Children and Adolescents

**DOI:** 10.3390/dj14050276

**Published:** 2026-05-07

**Authors:** Jonas Rafi, Magnus Hakeberg, Ulla Wide

**Affiliations:** 1 Department of Psychology, Stockholm University, Frescativägen 8, 106 91 Stockholm, Sweden; 2 Kronan Specialist Clinic, Landsvägen 39, Sundbyberg, 172 63 Stockholm, Sweden; 3 Institute of Odontology, Sahlgrenska Academy, University of Gothenburg, Medicinaregatan 12G, 413 90 Göteborg, Sweden; magnus.hakeberg@odontologi.gu.se (M.H.); ulla.wide@gu.se (U.W.)

**Keywords:** dental anxiety, children, adolescents, psychometrics, questionnaire, reliability, validity

## Abstract

**Background/Objectives**: Valid and reliable measurement of dental anxiety is of importance in both clinical and research settings. The aim of this study was to adapt and evaluate the Swedish child and adolescent version of the index of dental anxiety and fear (IDAF). **Methods**: A total of 142 dental patients aged 10–15 completed the four-components (IDAF-4C) and stimulus module (IDAF-S). Convergent validity was evaluated by correlating IDAF with CFSS-DS and a single item question on self-reported dental anxiety (SQDA). Reliability was investigated using Cronbach’s alpha, test–retest measurements (*n* = 16) and interviews. **Results**: IDAF-4C had a high correlation to CFSS-DS (r = 0.72). IDAF-4C convergent validity to SQDA (r = 0.76) was higher than the correlation between CFSS-DS and SQDA (r = 0.62). Convergent validity between IDAF-S and CFSS-DS was high (r = 0.83). Test–retest for IDAF-4C resulted in a moderate ICC of 0.72 (95% CI 0.37–0.89) and a correlation coefficient of 0.74. The difference in skipped items suggests that IDAF has higher usability than CFSS-DS. **Conclusions**: IDAF-4C provides a reliable estimate of dental anxiety, while IDAF-S provides clinical insights in individual aspects of dental anxiety similar to CFSS-DS. Further research includes evaluating the specific phobia module of IDAF and include more test–retest participants. The Swedish child and adolescent version of IDAF-4C and IDAF-S show good psychometric properties and usability and may be used as a dental anxiety measurement among children and adolescents.

## 1. Introduction

Dental anxiety may act as a barrier for dental visits and treatment by avoidance and other behavioural difficulties at the dental clinic. Thus, left untreated, dental anxiety may have serious implications on an individual’s oral health. Among children and adolescents, meta-analytic evidence suggests that 23.9% have dental anxiety [1]. However, the reported prevalence of dental anxiety is also affected by how it is measured and whether one uses a symptom-score cutoff or a diagnosis of specific phobia as criteria. When narrowing dental anxiety to only those who fulfil criteria for dental phobia or intra-oral injection phobia, a study of children and adolescents in Norway suggests prevalence estimates of 3.7% (95% CI: 2.7–5.1%) and 8.0% (95% CI: 6.5–9.9%), respectively [2]. Dental anxiety is preferably treated using cognitive behaviour therapy [3].

Similarly, the occurrence of dental anxiety differs by age group, such that preschool children have the highest occurrence (36.5%), followed by primary school children (25.8%) and teenagers (13.3%). Dental anxiety is also associated with other factors, such as gender and some sociodemographic determinants.

Several measurements exist that aim to measure dental anxiety among children and adolescents. A review of measures for dental anxiety in children found thirteen commonly used subjective measures [4]. These include the Children’s Fear Survey Schedule-Dental Subscale (CFSS-DS) and the modified dental anxiety scale (MDAS) [5,6,7]. Although showing good levels of reliability and validity, existing measures generally have a narrow focus on situational triggers that lack the multidimensional nature of anxiety and fear [8]. This means that the emotional, behavioural, cognitive, physical symptoms are not explored and measured, thus leading to an incomplete representation of dental fear [8,9].

The Index of Dental Fear and Anxiety (IDAF-4C+) was developed with an explicit theoretical underpinning that takes into account multiple components of fear and anxiety [8]. The IDAF-4C+ consists of three modules: a core module, a phobia module, and a stimulus module. The core module (IDAF-4C) measures cognitive, emotional, physiological, and behavioural components of dental anxiety. The phobia module (IDAF-P) includes questions that aid a clinician in assessing whether a DSM-IV [10] diagnosis is warranted. Lastly, the stimulus module (IDAF-S) measures anxiety towards ten common dental related stimuli.

The IDAF has been translated and culturally adapted to more than ten languages demonstrating overall high internal consistency reliability, test-rest reliability, and concurrent validity [11,12]. The Swedish version of IDAF-4C and IDAF-S show adequate psychometric properties, while IDAF-P may need further improvement [12,13]. However, just as a measurement need to be culturally adapted before using it in a new cultural context, an adaption of language and relevance is necessary before administrating the scale to children and adolescents. Such procedures has been done with the Malay and Turkish version of IDAF [14,15]. Here, age adaption from the adult version to the child and adolescent version included rephrasing of items to accommodate the fact that children and adolescents neither choose when to go to the dentists or make their appointments themselves.

To date, the Swedish version of the IDAF has yet to be age adapted to children and adolescents. The aim of the current study is to fill this important gap by age adapting IDAF-4C and IDAF-S to children and adolescents, explore its comprehension, and test the reliability and validity in a clinical setting.

## 2. Materials and Methods

### 2.1. Study Design and Participants

Data collection was performed at four clinics: two specialized pedodontic dental clinics and two general dental public service clinics in Sweden. A consecutive sample of participants were recruited by asking all Swedish speaking patients aged 10–15 to participate. Patients who agreed to participate received the study information, consent and measures on paper by a study coordinator at each clinic. Participants in one clinic were asked to fill in the questionnaire again after two weeks. The sample consisted of 142 participants (Table 1). Participant characteristics are shown in Table 1. For participants aged 10–14, informed consent was required from both the participant and one parent, whereas children aged 15 could participate on basis of their informed consent alone. Ethical approval was obtained by the Swedish Ethical Review Authority (Dnr: 2024-01744-01). No artificial intelligence has been used in any part of the analysis or writing.

### 2.2. Measurements

#### 2.2.1. Demographic Questions

The first part of the questionnaire consisted of questions regarding age, gender, education level of mother and father, and country of birth of mother and father.

#### 2.2.2. IDAF-4C and IDAF-S

The core module (IDAF-4C) and stimulus (IDAF-S) modules from IDAF-4C+ were included in the questionnaire. The Swedish version of IDAF-4C+ has shown to be a useful measure of dental anxiety in research and clinical practice [12]. However, as the phobia module may need further refinement and require additional clinical assessment for validation, it was omitted [13]. The core module includes eight questions covering cognitive, emotional, physiological, and behavioural components of anxiety, whereas the stimuli module includes ten questions. Both modules are scored on a 1–5 Likert scale. The core module asks to what extent one agrees to the questions, from “Disagree” to “Strongly Agree”, whereas the stimulus module queries the extent of worry, from “Not at all” to “Very much”.

#### 2.2.3. Single Question in Dental Anxiety (SQDA)

The SQDA consisted of the single question “Are you afraid of going to the dentist?” with the following response options scored 1–4: “Not at all”, “A little”, “Quite a lot”, and “Very much” [16].

#### 2.2.4. Children’s Fear Survey Schedule-Dental Subscale (CFSS-DS)

CFSS-DS is a dental specific measure of fear consisting of 15 items. Each item is scored 1–5, where the responses range from “Not afraid at all” to “Very afraid” [6].

### 2.3. Age Adaption Process

The child and adolescent versions of IDAF-4C and IDAF-S were adapted from the Swedish adult version of the IDAF-4C+. Two psychologists (JR, UW) and one dentist (MH) participated in an iterative adaption process, using clinical expertise in behavioural dentistry as well as previous adaptions processes to guide the procedure [14,15]. To take into account that children do not have full control of whether they will go to their dental visit or not, adaptions included changing phrases indicating control, such as changing “I postpone dental visits” to “I try to postpone dental visits”. Item g in IDAF-S (“I worry about the cost of dental treatment”) was removed since dental care in Sweden is free of charge for children and adolescents up to age 19 years. The first five participants who completed the data collection were asked to participate in a short interview to explore item comprehension. No changes were made following the interviews.

### 2.4. Data Analysis

Analyses were conducted in R (Version 4.5.0) [17] and IBM SPSS Statistics (Version 27).

For IDAF-4C, convergent validity was evaluated by examining Spearman’s rank-order correlations between both total and component scores on IDAF-4C and scores on SQDA and CFSS-DS. Reliability was evaluated by calculating Cronbach’s alpha, corrected item-total correlations, intercorrelation between IDAF-4C components and intercorrelation between IDAF-4C items. Test–retest reliability was examined by calculating Intraclass correlation coefficient between measurement occasions. For IDAF-S, convergent validity was evaluated by examining Spearman’s rank-order correlations between both total and item scores on IDAF-S with scores on IDAF-4C, SQDA and CFSS-DS. Reliability was evaluated by calculating Cronbach’s alpha, corrected item-total correlations, and intercorrelation between IDAF-4S. Construct validity was investigated using exploratory factor analysis using maximum likelihood extraction, oblimin rotation and eigenvalues greater than one. Analyses were conducted on observed data, meaning that no imputation was done on missing data. An a priori power analysis resulted in a required sample size of 128 participants for correlations of 0.25.

## 3. Results

### 3.1. Participant Characteristics

Participants demographic and clinical variables split by gender are presented in Table 1. A total of 142 participants were included in the study, comprising 83 boys and 58 girls. Missing data was observed: one participant had not answered the demographic questions, one had not answered the IDAF-S and CFSS questions, one had not answered any CFSS questions, and four participants had skipped specific items on the CFSS. The boys were slightly younger (12.3 years, SD = 1.7) than girls (12.9 years, SD = 1.6). Most participants (65.2%) reported no dental fear on the SQDA, whereas 22.7% reported being “a little” afraid, 8.5% “quite” afraid, and 3.5% “very” afraid. A higher proportion of boys (71.1%) than girls (56.9%) endorsed no fear, while girls more often reported higher levels of fear.

Mean scores on the IDAF-4C indicated generally low levels of dental fear (M = 1.4, SD = 0.7), with similar and non-significantly different scores across genders (*p* = 0.786), and the subscales (emotional, behavioural, physiological, and cognitive responses) showed comparable patterns. Participants also reported low levels of dental anxiety symptoms on the IDAF-S (M = 1.8, SD = 0.8), with girls showing slightly higher scores than boys. On the CFSS-DS, the overall mean total score was 23.2 (SD = 8.6), with girls (M = 24.2, SD = 8.2) scoring somewhat higher than boys (M = 22.6, SD = 8.9).

### 3.2. Validity of IDAF-4C

The Spearman correlations between IDAF-4C total score, IDAF-4C components, SQDA and CFSS-DS is shown it Table 2. The IDAF-4C was strongly correlated to CFSS-DS (r = 0.72) and SQDA (0.76), increasing evidence for convergent validity. Among the four components, correlations to the CFSS-DS ranged from 0.47 (IDAF-4C-B) to 0.66 (IDAF-4C-E). The corresponding correlations to the SQDA were similar but with larger variation, ranging from 0.42 (IDAF-4C-B) to 0.77 (IDAF-4C-E). The assumptions for EFA were met, indicated by a significant Bartlett’s Test of Sphericity, χ^2^ (28) = 885.78, *p* <  0.001 and KMO = 0.88. The 8 items loaded on one factor.

### 3.3. Reliability of IDAF-4C

Internal consistency of IDAF-4C was high, with a Cronbach’s alpha of 0.92. When removing single items, the alpha coefficient ranged from 0.90 to 0.92 thus indicating that the coefficient would not improve if items were removed. The corrected item-total correlations ranged from 0.68 (item 8) to 0.86 (item 3). Spearman correlations between the four components in IDAF-4C are shown in Table 2. The behavioural component had the lowest correlations to other components, ranging from 0.26 to 0.39. The intercorrelations among the other components ranged from 0.55 to 0.68. Intercorrelations between specific items ranged from 0.15 (items 2 and 6) to 0.69 (items 1 and 3). Sixteen participants completed the IDAF-4C twice for the test–retest analysis. The test–retest analysis resulted in a moderate ICC of 0.72 (95% CI 0.37–0.89) and a correlation coefficient of 0.74.

### 3.4. Validity of IDAF-S

The Spearman correlations between IDAF-S total score, SQDA and CFSS-DS is shown in Table 2, and the item-specific correlations are shown in Table 3. There was a strong correlation between IDAF-S and CFSS-DS (r = 0.83), and a moderate correlation between IDAF-S and SQDA (r = 0.57). Correlations between IDAF-S and the four components in IDAF-4C was highest for IDAF-4C-E (r = 0.88), followed by IDAF-4C-P (r = 0.79), IDAF-4C-C (r = 0.76), and lastly IDAF-4C-B (0.53). Analyzing item-specific correlations, it was found that “Having control” (item 3, r = 0.6) had the highest correlation with SQDA, while “Embarrassment” (item 2, r = 0.3) had the lowest correlation. “Fear of pain” (item 1, r = 0.67) had the highest correlation with IDAF-4C, while “Embarrassment” (item 2, r = 0.27) had the lowest. The assumptions for EFA were met, indicated by a significant Bartlett’s Test of Sphericity, χ^2^ (36) = 4956.76, *p* <  0.001 and KMO = 0.97. The 9 items loaded on one factor.

### 3.5. Reliability of IDAF-S

Internal consistency of IDAF-4C was high, with a Cronbach’s alpha of 0.88. When removing single items, the alpha coefficient ranged from 0.85 to 0.89. Item 2 (“shame or embarrassment”) was the only item that indicated improvement of the scale if removed by increasing Cronbach’s alpha from 0.88 to 0.89. Item 2 also had the lowest corrected item-total correlations (0.36), while the others ranged from 0.55 (item 7) to 0.79 (item 6). Removing item 2 increased the range of corrected item-total correlations for the remaining items from 0.59 to 0.85. Intercorrelations between specific items ranged from 0.07 (item 2 with items 5 and 7) to 0.69 (items 3 and 6).

## 4. Discussion

The current study adapted the Swedish version of two IDAF-4C+ modules, the IDAF-4C and IDAF-S, for use with children and adolescents. Thereafter, aspects of validity and reliability was explored. The adaption process included removing one item (“worry about cost of dental visits”) since dental care is currently free of charge for children and adolescents in Sweden. Therefore, when comparing the child and adolescent version of IDAF-S to the adult version, it is important to consider that means rather than total scores are used.

Comprehension was explored by asking a subset of respondents if any items were unclear. No IDAF items needed explanation. However, there were some questions about the CFSS-DS, mainly regarding what is meant by “having the nurse clean your teeth”. Furthermore, *n* = 4 (3%) skipped at least one question in the CFSS-DS questionnaire, as compared to zero skipped items in the IDAF questionnaire. This could imply that the IDAF is easier to understand and respond to compared to the CFSS-DS.

The convergent validity of IDAF-4C to CFSS-DS and SQDA was high (0.72 and 0.76, respectively). Previously, the validity and reliability of the adult version of IDAF have been explored for regular dental patients and patients with severe dental anxiety [12,13]. Of the participants in the current study, 12% reported being more than a little anxious of dentistry. The IDAF-4C mean scores (M = 1.4, SD = 0.7), are more comparable to the regular dentist patients in Wide Boman et al. (M = 1.6, SD = 0.9) than the patients with severe dental anxiety in Svensson et al. (M = 3.9, SD = 0.7) [12,13]. Convergent validity in Wide Boman et al. was 0.86 to DFS and 0.87 to SQDA, whereas the correlations ranged for different scales ranged from 0.69 to 0.75 in Svensson et al. [12,13]. Still, the correlation between IDAF-4C and SQDA was higher than the correlation between CFSS-DS and SQDA. Thus, the IDAF-4C convergent validity can be considered high and with a higher correlation to self-assessed dental anxiety than the CFSS-DS. Cronbach’s alpha for IDAF-4C was high (0.92), which may indicate redundant items. However, the Cronbach’s alpha of some other language versions are also high, more than 0.90 [8,18,19], suggesting that the risk of redundancy is present in other versions as well.

Looking at the specific components of IDAF-4C, the emotional component correlates the most to CFSS-DS and SQDA, as well as to the IDAF-4C total score. This is in line with previous studies [8,12]. In contrast, the behaviour component had the lowest correlation to other measures, whereas cognition had the lowest correlation in the aforementioned studies. One reason could be that children and adolescents have lower control to decide if and when they should go to the dentist. One possibility is that children and adolescents show more avoidance behaviours at the clinic rather than before the appointment to the clinic. Previous research has shown that children with both internalizing and externalizing problems have a higher prevalence of negative behaviours, such as uncooperative behaviour, during dental treatment [20]. Future research may investigate whether there are more relevant behaviours among children and adolescents that capture dental anxiety and fear than avoiding, delaying and postponing dental visits. One possibility would be to phrase one behavioural item more generally (e.g., “refusing to cooperate”) in order to catch as many relevant behaviours as possible, such as refusing to sit in the chair, being silent, having angry outbursts, or refusing to open the mouth.

The stimulus module of IDAF-S correlated strongly to CFSS-DS (0.83) and moderately to SQDA (0.57). The strong correlation to CFSS-DS is not surprising as both the CFSS-DS and IDAF-S queries responses to stimuli that may trigger dental anxiety. The moderate correlation with the SQDA shows that dental anxiety is better captured with IDAF-4C than IDAF-S. Ideally, the IDAF-S could be used as clinical guidance when assessing triggers. Looking at the specific items of IDAF-S, it is shown that items 1 (pain or discomfort), 3 (lack of control), and 6 (not knowing what will happen) correlates highest to both SQDA and IDAF-4C. Interestingly, these stimuli are larger indicators of dental anxiety than fear of needles. However, fear of needles was the item with highest mean score. Similar results were found in the adult version, although with a higher correlation between dental anxiety and item 10 (unkind dentist). Item 2 (shame or embarrassment) was shown to have lower corrected item-total correlation and intercorrelation than other items. Although this study is not designed to create explanations for this finding, further studies could explore whether shame and embarrassment in a dental settings could be viewed as a facet of dental anxiety, a dental specific symptom not belonging to the dental anxiety construct (e.g., social anxiety), or a domain unrelated to anxiety. Shame in dental settings is a complex but underexplored phenomenon influenced by personal, social, economic, cultural and systemic factors [21]. However, embarrassment and shame before a dental visit is a clinically relevant observation regardless of the underlying construct. It is important for clinicians to identify and further explore symptoms, such as shame, that may maintain behavioural problems in the dental settings. Still, future studies may investigate whether this item is correlated with the social anxiety item in IDAF-P, trauma, or something else, and whether the item should be retained in IDAF-S or better used in another way.

The study is not without limitations. While not evaluated here, the IDAF has a third module which aims to identify specific phobia (IDAF-P) and distinguish it from general anxiety, panic disorder and social phobia. However, as the Swedish adult version of IDAF-P may need more development, it was not adapted to children and adolescents [13]. Future research may improve and adapt IDAF-P to children and adolescents and compare it to structured clinical interviews. A second limitation is the use of a consecutive sampling design. Although it may introduce biases due to the non-probability technique, it reduces selection biases and is time- and cost-efficient. A third limitation is the low sample in the test–retest analyses, leading to unstable and imprecise estimates. A fourth limitation is the lack of discriminant validity and known groups validity. A comprehensive psychometrical validation using clinician assessment of dental phobia and IDAF-P would extend the validation procedure, preferably with more participants with high dental anxiety.

## 5. Conclusions

In summary, the child and adolescent version of the Swedish IDAF-4C and IDAF-S showed good psychometric properties with moderate to strong convergent validity with other relevant measures as well as good reliability and was perceived as easy to use. Together with the flexible use of modules, the results of this study increase the evidence for the IDAF as a novel and useful measure for dental anxiety measurement. However, as with all measurements there is room for improvement, such as calibrating the behaviour domain of the IDAF-4C to better correspond to overall dental anxiety.

## Figures and Tables

**Table 1:** Participant age and mean scores on IDAF-4C, its components, IDAF-S, SQDA, and CFSS-DS.

	Overall *n* = 142	Boys *n* = 83	Girls *n* = 58
Age	12.6 (1.7)	12.4 (1.7)	12.9 (1.6)
SQDA ^1^			
No	92 (65.2%)	59 (71.1%)	33 (56.9%)
Yes, a little	32 (22.7%)	17 (20.5%)	15 (25.9%)
Yes, quite	12 (8.5%)	6 (7.2%)	6 (10.3%)
Yes, very	5 (3.5%)	1 (1.2%)	4 (6.9%)
IDAF-4C ^2^	1.4 (0.7)	1.4 (0.7)	1.5 (0.7)
IDAF-4C-C ^3^	1.4 (0.7)	1.4 (0.7)	1.4 (0.8)
IDAF-4C-E ^4^	1.6 (0.9)	1.5 (0.8)	1.7 (1.0)
IDAF-4C-P ^5^	1.5 (0.8)	1.4 (0.8)	1.6 (0.9)
IDAF-4C-B ^6^	1.3 (0.9)	1.4 (0.9)	1.2 (0.8)
IDAF-S ^7^	1.8 (0.8)	1.7 (0.8)	1.9 (0.8)
CFSS-DS ^8^	23.2 (8.6)	22.6 (8.9)	24.2 (8.2)

Note. Values are presented as *n* (%) or mean (SD). ^1^ SQDA; single-question assessment of dental anxiety; ^2^ IDAF-4C, index of dental anxiety and fear (four-components module); ^3^ IDAF-4C-C, cognitive component; ^4^ IDAF-4C-E, emotional component; ^5^ IDAF-4C-P, physiological component; ^6^ IDAF-4C-B, behavioural component; ^7^ IDAF-S, index of dental anxiety and fear stimulus module; ^8^ CFSS-DS, Children’s Fear Survey Schedule-Dental Subscale.

**Table 2:** Spearman correlations between the mean IDAF-4C score, its components, IDAF-S, CFSS-DS and single-question assessment of dental anxiety (SQDA).

	1	2	3	4	5	6	7
1. IDAF-4C ^1^							
2. IDAF-4C-C ^2^	0.76						
3. IDAF-4C-E ^3^	0.88	0.61					
4. IDAF-4C-P ^4^	0.79	0.55	0.68				
5. IDAF-4C-B ^5^	0.53	0.39	0.35	0.26			
6. IDAF-S ^6^	0.72	0.58	0.63	0.6	0.41		
7. CFSS-DS ^7^	0.72	0.57	0.66	0.54	0.47	0.83	
8. SQDA ^8^	0.76	0.61	0.77	0.67	0.42	0.57	0.62

Note. Values are presented as *n *(%) or mean (SD). ^1^ IDAF-4C, index of dental anxiety and fear (four-components module); ^2^ IDAF-4C-C, cognitive component; ^3^ IDAF-4C-E, emotional component; ^4^ IDAF-4C-P, physiological component; ^5^ IDAF-4C-B, behavioural component; ^6^ IDAF-S, index of dental anxiety and fear stimulus module; ^7^ CFSS-DS, Children’s Fear Survey Schedule-Dental Subscale; ^8^ SQDA; single-question assessment of dental anxiety.

**Table 3:** Descriptive statistics for IDAF-S and correlations to IDAF-4C and SQDA.

Item	Mean (SD)	Correlation with SQDA	Correlation with IDAF-4C
1. Pain	2.19 (1.16)	0.55	0.67
2. Embarrassment	1.17 (0.57)	0.30	0.27
3. Control	1.64 (1.13)	0.60	0.63
4. Disgust	1.56 (0.93)	0.34	0.43
5. Anesthesia	1.78 (1.16)	0.38	0.46
6. Unknown agenda	1.67 (1.13)	0.50	0.60
7. Needles	2.82 (1.51)	0.32	0.46
8. Vomiting	1.58 (1.03)	0.36	0.44
9. Disagreeable	1.50 (1.11)	0.36	0.44

Note. SQDA, single-question assessment of dental anxiety; IDAF-4C, index of dental anxiety and fear (four-components module); SD, standard deviation.

## Data Availability

The data presented in this study are available on request from the corresponding author. The data underlying this study are not publicly available because they include sensitive information concerning children’s health, the disclosure of which is restricted by ethical and legal considerations.

## Abbreviations

The following abbreviations are used in this manuscript:

SQDA	Single-question assessment of dental anxiety
IDAF-4C	index of dental anxiety and fear (four-components module)
IDAF-4C-C	cognitive component
IDAF-4C-E	emotional component
IDAF-4C-P	physiological component
IDAF-4C-B	behavioural component
IDAF-S	Index of dental anxiety and fear stimulus module
CFSS-DS	Children’s Fear Survey Schedule-Dental Subscale
